# The impact of severity of hypertension on association of PGC-1*α *Gene with blood pressure and risk of hypertension

**DOI:** 10.1186/1471-2261-7-33

**Published:** 2007-10-31

**Authors:** Gaoqiang Xie, Dongshuang Guo, Ying Li, Shengying Liang, Yangfeng Wu

**Affiliations:** 1 Department of Epidemiology, Cardiovascular Institute and Fuwai Hospital, Chinese Academy of Medical Sciences and Peking Union Medical College, The National Center for Cardiovascular Disease Control and Research, China; 2 Department of Cardiovascular Epidemiology, Yu Xian Renmin Hospital, Shanxi, China; 3 Department of Epidemiology and Biostatistics, Peking University School of Public Health, Beijing, China; 4 The George Institute, China, Beijing, China

## Abstract

**Background:**

Little is known about the impact of severity of hypertension on the association of genes with high blood pressure, which may cause the inconsistently reported associations of peroxisome proliferator-activated receptor-*γ *coactivator-1*α *(PGC-1*α*) gene with blood pressure.

**Methods:**

A cardiovascular epidemiology survey and genotyping were performed in a population-based sample of 1642 apparently healthy residents (648 men and 994 women aged 35–91 years).

**Results:**

After adjusting for age, sex, body mass index, and antihypertensive medication, G482S and +2962A/G polymorphisms were significantly associated with systolic blood pressures in hypertension patients with medication use (p = 0.023 and 0.022 for G482S and +2962A/G respectively) but not in all participants, normotensives, and patients with no medication use. Multivariable logistic models showed that the two polymorphisms were significantly associated with severe hypertension (SBP ≥ 160 mm Hg or DBP ≥ 100 mm Hg regardless of medication use), with an OR of 0.6(95% confidence interval [CI]: 0.4–0.98) for S482S vs. G482G and an OR of 1.9(95% CI: 1.2–3.0) for +2962G/G vs. +2962A/A, but not with regular hypertension (SBP ≥ 140 mm Hg or DBP ≥ 90 mm Hg or current use of antihypertensive medications), with an OR of 0.9(95% CI: 0.7–1.2) for S482S vs. G482G and an OR of 0.9(95% CI: 0.7–1.4) for +2962G/G vs. +2962A/A. Haplotype combination analyses showed a significant synthetic effect (OR of severe hypertension for persons with G482X and +2962G/G = 2.6, 95%CI: 1.5–4.4, with reference to persons with S482S and +2962A/X).

**Conclusion:**

In this study, we found that G482S and +2962A/G polymorphisms of PGC-1*α *gene were only significantly associated with severe hypertension defined by occasional clinic blood pressure measurements. This finding suggested severe hypertension rather than regular hypertension should be used as the outcome in studies on association of genes with blood pressure or hypertension, in order to have a better power.

## Background

Hypertension is the most common risk factor for cardiovascular and cerebrovascular diseases and affected over 1 billion individuals worldwide[1,2]. Although some risk factors of hypertension such as high sodium intake, inactivity, obesity, familial history, etc. have been identified, genetic factors of hypertension at gene level remain unclear. This is possibly partly due to so called "minor effect" of the genes[3]. Also, it is possibly due to dilution effect from environment factors because of using a lower cut off of blood pressure to define hypertension cases. However, few studies have examined this hypothesis.

Peroxisome proliferator-activated receptors gamma coactivator 1 alpha (PGC-1*α*, Gene ID, No. 10891) is a coactivator of several nuclear receptors including peroxisome proliferator-activated receptors *α *and *γ*, thyroid hormone receptor, mineral corticoid receptor and estrogen receptors, and was considered involving in blood pressure regulation [4,5]. Recently, several western population studies have studied the associations of Gly482Ser (G482S) and +2962A/G single nucleotide polymorphisms (SNP) of PGC-1*α *gene with blood pressure [6-10]. However, the associations were inconsistent in different studies, even in the same ethnic populations. Moreover, the association has not been studied in Asian populations.

The aim of this study was to investigate whether the G482S and +2962A/G polymorphisms in the PGC-1*α *gene were associated with blood pressure and the impact of severity of hypertension on the association of PGC-1*α *Gene with blood pressure and risk of hypertension in a middle-aged and elderly population in China.

## Methods

### Study population

We drew our study participants from 10 villages in a northern Chinese population in 2004. To be recruited in the study, the participant must be a registered resident and living in the village in the year of survey. Among 8217 registered residents, 2835 met the inclusion criteria of ≥35 years of age, and out of them 2409 were living in the village at the year of survey and thus invited. Among the invited, 1998 accepted to participate and gave their informed consent; and 1701 had their genotypic information. We excluded 59 patients with myocardial infarction and stroke from analyzed population. We also excluded 5 participants with missing data of blood pressure. Finally 1642 participants were included in our analyses. The study was approved by the Ethical Committee of Fuwai Hospital according to the Declaration of Helsinki. The written informed consent was obtained from all participants.

### Blood Pressure Measurement

Three BP measurements were obtained from each participant using a standard mercury sphygmomanometer by trained and certified observers, according to a standard protocol described in detail elsewhere[11,12]. The average of the 3 blood pressure readings were used to define hypertension and for analysis in the present paper. Current use of antihypertensive medication was determined by a "yes" or "no" answer to the Chinese equivalent of the question, "Have you taken antihypertensive drugs in the past 2 weeks?"

To explore the impact of severity of hypertension on the association between the gene and hypertension (or blood pressure), we used different cut offs to define hypertension. First, we defined hypertension using the current widely accepted criteria [2] as SBP ≥ 140mmHg or DBP ≥ 90 mmHg or current use of antihypertensive medications. We referred it as "regular hypertension" in this paper. Then, we also defined hypertension as SBP ≥ 160mmHg or DBP ≥ 100mmHg regardless of using antihypertensive medication. We referred it as "severe hypertension" in this paper.

### Body Mass Index (BMI)

Body height was measured to the nearest centimeter (cm) using a standard right-angle device. Body weight was measured to the nearest kilogram by using a spring balance. Each participant was measured in typical indoor clothing, without shoes, and in a standing position. BMI was defined as weight/height^2 ^(kg/m^2^).

### Genotyping

We genotyped the G482S (refSNP ID: rs8192678) and +2962A/G (refSNP ID: rs6821591) polymorphisms in the PGC-1*α *gene using DNA MassARRAY Technology[13,14]. Briefly, a fragment (approximately 100bp) containing the SNP site was amplified by PCR first. All PCR reactions were performed on an ABgene (#TF-0384), 384 well PCR plate. The sample plate and a 384 SpectroCHIP were placed on the deck of the SpectroPOINT robot. The robot transferred a few nanolitres of solution from the sample plate onto the chip and calibrant was transferred from reservoir onto calibrant patches of 384 SpectroCHIP. The chip was read in the Bruker Autoflex Mass Spectrometer system.

### Statistical Analysis

SHEsis software[15] was used for Hardy-Weinberg equilibrium test, haplotype construction, and linkage analyses. Chi-square test (*χ*^2^) and odds ratio (OR) test were used for dichotomous variables. Logistic regression and ANOVA were used for adjustment for multiple covariates including sex, age, body mass index (BMI), and antihypertensive treatment, using Version 9.0 of SAS system for Windows. A p-value less than 0.05 was considered statistically significant. Statistical power was calculated according to the following formula.

Z1−β=n(p1−p2)−Z1−α22p(1−p)p1(1−p1)+p2(1−p2)
 MathType@MTEF@5@5@+=feaafiart1ev1aaatCvAUfKttLearuWrP9MDH5MBPbIqV92AaeXatLxBI9gBaebbnrfifHhDYfgasaacH8akY=wiFfYdH8Gipec8Eeeu0xXdbba9frFj0=OqFfea0dXdd9vqai=hGuQ8kuc9pgc9s8qqaq=dirpe0xb9q8qiLsFr0=vr0=vr0dc8meaabaqaciaacaGaaeqabaqabeGadaaakeaacqqGAbGwdaWgaaWcbaGaeeymaeJaeyOeI0ccciGae8NSdigabeaakiabg2da9maalaaabaWaaOaaaeaacqqGUbGBaSqabaGccqGGOaakcqqGWbaCdaWgaaWcbaGaeeymaedabeaakiabgkHiTiabbchaWnaaBaaaleaacqqGYaGmaeqaaOGaeiykaKIaeyOeI0IaeeOwaO1aaSbaaSqaaiabbgdaXiabgkHiTmaaliaabaGae8xSdegabaGaeeOmaidaaaqabaGcdaGcaaqaaiabbkdaYiabbchaWjabcIcaOiabbgdaXiabgkHiTiabbchaWjabcMcaPaWcbeaaaOqaamaakaaabaGaeeiCaa3aaSbaaSqaaiabbgdaXaqabaGccqGGOaakcqqGXaqmcqGHsislcqqGWbaCdaWgaaWcbaGaeeymaedabeaakiabcMcaPiabgUcaRiabbchaWnaaBaaaleaacqqGYaGmaeqaaOGaeiikaGIaeeymaeJaeyOeI0IaeeiCaa3aaSbaaSqaaiabbkdaYaqabaGccqGGPaqkaSqabaaaaaaa@5C40@

In this formula, n is the total number of participants, p is total prevalence of hypertension, p_1 _and p_2 _are prevalence of hypertension in two compared genotypes groups, *α *= 0.05 was permitted as possibility of type I errors (Z_1-*α*/2 _= 1.96). Then Z_1-*β *_is calculated and statistical power is estimated.

The difference of ORs was tested using Z-test according to the following formula.

Z=ln⁡OR1−ln⁡OR2σln⁡OR12+σln⁡OR22
 MathType@MTEF@5@5@+=feaafiart1ev1aaatCvAUfKttLearuWrP9MDH5MBPbIqV92AaeXatLxBI9gBaebbnrfifHhDYfgasaacH8akY=wiFfYdH8Gipec8Eeeu0xXdbba9frFj0=OqFfea0dXdd9vqai=hGuQ8kuc9pgc9s8qqaq=dirpe0xb9q8qiLsFr0=vr0=vr0dc8meaabaqaciaacaGaaeqabaqabeGadaaakeaacqWGAbGwcqGH9aqpdaWcaaqaaiGbcYgaSjabc6gaUjabd+eapjabdkfasnaaBaaaleaacqaIXaqmaeqaaOGaeyOeI0IagiiBaWMaeiOBa4Maem4ta8KaemOuai1aaSbaaSqaaiabikdaYaqabaaakeaadaGcaaqaaGGaciab=n8aZnaaDaaaleaacyGGSbaBcqGGUbGBcqWGpbWtcqWGsbGucqaIXaqmaeaacqaIYaGmaaGccqGHRaWkcqWFdpWCdaqhaaWcbaGagiiBaWMaeiOBa4Maem4ta8KaemOuaiLaeGOmaidabaGaeGOmaidaaaqabaaaaaaa@4F46@

## Results

### General characteristics of participants

Table 1 showed general characteristics of the participants. Briefly, 648 men and 994 women participated in the study. The age varied from 35 to 91 years and was 53 years old on average. The prevalence was 48% for regular hypertension and 16% for severe hypertension. The severe hypertension accounted for 33.7% of the regular hypertension. 56.6% of regular hypertension and 59.3% of severe hypertension used antihypertensive medications in the past 2 weeks (Table 1).

**Table 1:** Characteristics of study population

Variables	Values
No. of participants	1642
% of men (No.)	39.46%(648)
Age, years	52.8(10.2)
BMI, kg/m^2^	24.2(3.7)
SBP, mmHg	134.9(21.8)
DBP, mmHg	82.9(10.9)
Regular hypertension*	48.4%(795)
Severe hypertension**	16.3%(268)
Antihypertensive medication use,%	27.41%(450)
Antihypertensive medication use among regular hypertension,%	56.6%(450)
Antihypertensive medication use among severe hypertension,%	59.3%(159)
Gly482Ser genotypes	
S482S	19.24%(316)
G482S	49.63%(815)
G482G	31.12%(511)
+2962A/G genotypes	
+2962A/A	51.16%(840)
+2962A/G	39.04%(641)
+2962G/G	9.81%(161)
Haplotype combination	
S482S||+2962A/A	19.0% (312)
S482S||+2962A/G	0.2% (4)
S482S||+2962G/G	0(0)
G482S||+2962A/A	23.9% (392)
G482S||+2962A/G	25.6% (421)
G482G||+2962A/A	8.3% (136)
G482G||+2962A/G	13.2% (216)
G482S||+2962G/G	0.1% (2)
G482G||+2962G/G	9.7% (159)

Data are presented as mean(SD) or %(n);

* Regular hypertension: SBP ≧ 140 mm Hg or DBP ≧ 90mm Hg or current use of antihypertensive medications;

** Severe hypertension: SBP ≧ 160 mm Hg or DBP ≧ 100mm Hg, regardless of medication use.

### Distribution of G482S and +2962A/G genotypes

The frequencies of wild-type (G482G), heterozygous (G482S), and variant genotypes (S482S) were 0.311, 0.496, and 0.192. The frequencies of wild-type (+2962A/A), heterozygous (+2962A/G), and variant genotypes (+2962G/G) were 0.512, 0.390, and 0.098 respectively. The distributions were consistent with Hardy-Weinberg equilibrium (HW-E) for G482S polymorphisms (p = 0.780) but not for +2962A/G (p = 0.018). In addition, the wild-type and variant allele frequencies for G482S polymorphism were 0.559 and 0.441. Respective frequencies for the +2962A/G were 0.707 and 0.293. Estimated haplotype frequencies were 0.269, 0.291, 0.438, and 0.002 for the double-wild-type allele (482G/+2962A), the allele with the variant nucleotide at +2962(482G/+2962G), the allele with the variant nucleotide at G482S (482S/+2962A), double-variant allele (482S/+2962G), respectively. There were no significant differences between men and women in genotype, allele, or estimated haplotype frequencies.

### Linkage analyses of G482S and +2962A/G

There was significant linkage disequilibrium between the +2962A/G and G482S polymorphism (D' = 0.981, *p *< 0.001). The distribution of the +2962A/G polymorphism was significantly associated with the distribution of the G482S polymorphism. The percentage of carriers of +2962 G/G were 0%,0.3%, and 31.1% among carriers of S482S, G482S, and G482G genotypes, and the corresponding percents of carriers of the +2962A/A genotype were 98.7%,48.1%, and 26.6% (p < 0.0001).

### Association of G482S and +2962A/G to blood pressure

We compared blood pressure among genotype groups. After adjustment for age, sex, body mass index, and current use of antihypertensive medication, SBP and DBP were not significantly associated with G482S genotypes (p = 0.266) or +2962A/G genotypes (p = 0.103) (Table 2). Sex-specific analyses showed similar results (data not shown in table). In order to exclude the confounding effect of antihypertensive medication, we analyzed the associations in three subgroups: 1) normal blood pressure free from current use of antihypertensive medications; 2) hypertension free from current use of hypertensive medications; 3) current use of antihypertensive medications. We found a significant association of SBP to G482S genotypes (p = 0.023) and +2962A/G genotypes (p = 0.020) in subgroup 3 but not in subgroup 1 or 2 (Table 3).

**Table 2:** Muti-variable-adjusted means of blood pressure (mm Hg) and standard errors by Gly482Ser and +2962A/G polymorphisms in 1642 participants

Genotypes	N	SBP(mm Hg)	DBP(mm Hg)
G482S genotypes			
G482G	511	139.2(0.9)	85.2(0.5)
G482S	815	139.4(0.7)	84.7(0.4)
S482S	316	137.4(1.1)	84.6(0.6)
p values		0.266	0.630
+2962A/G genotypes			
+2962A/A	840	138.8(0.7)	84.7(0.4)
+2962A/G	641	138.6(0.8)	84.9(0.4)
+2962G/G	161	141.3(1.5)	85.4(0.8)
p values		0.103	0.695

Note: values were adjusted for age, sex, BMI, and current use of antihypertensive medications.

**Table 3:** Muti-variable-adjusted means of blood pressure (mm Hg) among Gly482Ser and +2962A/G polymorphism in three subgroups

Genotypes	Normotensives			Hypertensive free from current antihypertensive medication			Current use of antihypertensive medication		
			
	N	SBP(mm Hg)	DBP(mm Hg)	N	SBP(mm Hg)	DBP(mm Hg)	N	SBP(mm Hg)	DBP(mm Hg)
G482S genotypes									
G482G	250	119.7(0.6)	77.7(0.4)	86	150.7(1.4)	90.2(0.9)	155	151.2(1.9)	89.0(1.0)
G482S	428	120.1(0.5)	77.2(0.3)	155	149.6(1.1)	90.8(0.7)	209	152.6(1.6)	88.4(0.8)
S482S	169	121.3(0.7)	78.2(0.5)	61	148.6(1.8)	90.6(1.2)	86	144.8(2.5)	87.0(1.3)
p values		0.207	0.286		0.647	0.897		0.023	0.426
+2962A/G genotypes									
+2962A/A	429	120.5(0.5)	77.6(0.3)	179	149.6(1.1)	90.9(0.7)	232	149.2(1.5)	87.4(0.8)
+2962A/G	336	120.1(0.5)	77.6(0.4)	127	150.2(1.3)	90.0(0.8)	178	151.0(1.8)	89.4(0.9)
+2962G/G	82	119.4(1.1)	77.2(0.7)	39	149.6(2.3)	91.0(1.5)	40	159.9(3.6)	89.4(1.9)
p values		0.580	0.899		0.930	0.667		0.020	0.181

Means and p values were adjusted for age, sex, and BMI. Values in () are standard errors.

### Association of G482S and +2962A/G to hypertension

The prevalence of regular hypertension varied but did not differ among genotype groups for both G482S and +2962A/G. Adjustment for sex, age, body mass index did not change the results (Table 4).

**Table 4:** Prevalence and odds ratios (ORs) of regular and severe hypertension by PGC-1*α *polymorphisms in 1642 participants

		Regular hypertension*			Severe hypertension**			
				
Genotypes	Total No.	Cases	Prevalence,%	OR_1_(95%CI)	Cases	Prevalence,%	OR_2_(95%CI)	p values for OR_1 _vs. OR_2_
G482S genotypes								
G482G	511	261	51.1%	1.0	91	17.8%	1.0	
G482S	815	387	47.5%	0.9(0.7–1.1)	139	17.1%	1.0(0.8–1.4)	0.503
S482S	316	147	46.5%	0.9(0.7–1.2)	38	12.0%	0.6(0.4–0.98)	0.177
p values			0.335	0.667		0.067	0.064	
+2962A/G genotypes								
+2962A/A	840	411	48.9%	1.0	128	15.2%	1.0	
+2962A/G	641	305	47.6%	0.9(0.7–1.1)	102	15.9%	1.1(0.8–1.4)	0.407
+2962G/G	161	79	49.1%	0.9(0.7–1.4)	38	23.6%	1.9(1.2–3.0)	0.015
p values			0.863	0.623		0.030	0.011	

* Regular hypertension: SBP ≧ 140 mm Hg or DBP ≧ 90mm Hg or current use of antihypertensive medications;

** Severe hypertension: SBP ≧ 160 mm Hg or DBP ≧ 100mm Hg, regardless of medication use.

OR_1_: adjusted for sex, age, BMI.

OR_2_: adjusted for sex, age, BMI, and antihypertensive medication.

P values for prevalence were from Chi-square test, p values for trend of ORs were from logistic regression, and p values for OR_1 _vs. OR_2 _were from Z-test.

The prevalence of severe hypertension differed at borderline significance among genotype groups for G482S and significantly for +2962A/G. After adjustment for age, sex, body mass index, and current use of antihypertensive medications, risk of severe hypertension was significantly lower for S482S in comparison with that for G482G (OR = 0.6; 95% confidence interval [CI]: 0.4–0.98) and significantly higher for +2962G/G than that for +2962A/A (OR = 1.9, 95% CI: 1.2–3.0). Sex-specific OR for +2962G/G vs. +2962A/A were 2.2(95%CI: 1.03–4.5) for men and 1.9(95%CI: 1.1–3.2) for women. However, sex-specific OR for G482S polymorphisms was no longer significant (OR = 0.6, 95%CI: 0.4–1.1 for men, OR = 0.7, 95%CI: 0.4–1.1 for women for S482S vs. G482G).

### Association of G482S and +2962A/G haplotype combination to hypertension

According to results from Table 4, we grouped all possible combinations of haplotype into 4 groups as shown in Table 5. The results clearly showed a synthetic effect of G482S and +2962A/G in developing severe hypertension but not for regular hypertension.

**Table 5:** Haplotype combination analyses of G482S and +2962A/G for regular and severe hypertension in 1642 participants

		Regular hypertension*			Severe hypertension**			
				
Haplotype combinations	N	Cases	Prevalence,%	OR_1_(95%CI)	Cases	Prevalence,%	OR_2_(95%CI)	p values for OR_1 _vs. OR_2_
S482S||+2962A/X	316	147	46.5%	1	38	12.0%	1.0	
S482S||+2962G/G	0	0	0		0	0		
G482X||+2962A/X	1165	569	48.8%	1.0(0.8–1.3)	192	16.5%	1.5(1.0–2.2)	0.131
G482X||+2962G/G	161	79	49.1%	1.0(0.7–1.5)	38	23.6%	2.6(1.5–4.4)	0.006
p values			0.753	0.989		0.005	0.002	

* Regular hypertension: SBP ≧ 140 mm Hg or DBP ≧ 90mm Hg or current use of antihypertensive medications;

** Severe hypertension: SBP ≧ 160 mm Hg or DBP ≧ 100mm Hg, regardless of medication use.

OR_1_: adjusted for sex, age, BMI.

OR_2_: adjusted for sex, age, BMI, and antihypertensive medication.

X was used to represents all possible allele, e.g. +2962A/X stands for +2962A/A or +2962A/G.

P values for prevalence were from Chi-square test, p values for trend of ORs were from logistic regression, and p values for OR_1 _vs. OR_2 _were from Z-test.

### Post hoc analysis of power

To understand whether our study has enough statistical power to conclude, we did analysis of power using the number of participants, prevalence of hypertension, and permitted possible type I error. The results showed that we had a power of 90% in analysis for the association of G482S genotype to the risk of severe hypertension, but it was only 46% for regular hypertension. For +2962A/G, we had 99% power for severe hypertension and only 4% for regular hypertension. For haplotype combination, we had 99% power for severe hypertension and only 14% for regular hypertension.

## Discussion

Our study has a number of strengths. First, its study population was community-based. Secondly, International standardized methodology and quality control procedures were used, as part of the China Multicenter Collaborative Study of Cardiovascular Epidemiology[16]. Thirdly, genotyping was conducted in the Beijing Genomics Institute Life Tech Co. Ltd. which was one of genotyping centres for the International HapMap Consortium [17]. Theses gave us a better opportunity to detect the true association of our interested genotypes to hypertension.

In our study, G482S and +2962A/G polymorphisms and their haplotypes were found significantly associated with risk of sever hypertension but not with that of regular hypertension. These findings were confirmed by our findings that these two SNPs were significantly associated with blood pressure in patients using antihypertensive medications but not in all participants, normotensives or patients with no medication, and were independent of age, sex, body mass index, and antihypertension medication use. These findings are well in accordance with the current knowledge of hypertension that the disease is caused by not only genetic factors but also environment factors[3]. It is those who exposed to both genetic and environment factors develops severe hypertension and thus had a better power to detect the effect of gene, while those who exposed to only environment factors or genetic factors develops light or moderate hypertension, which should be mostly composed of those caused by environment factors because of the much higher prevalence of environment factors, and it is hardly to detect the effect of gene among them. The dilution effect from environment factors plus the gene's "minor effect" makes it impossible or very hardly to detect the effect of gene by using a lower cut off to define hypertension. Otherwise, a very large sample of more than tens or hundreds thousands participants is needed to make up, as shown by our post hoc analysis of power. As we knew, this is the first study found that PGC-1*α *gene variants had stronger associations with more severe hypertension. These findings have very important significance of generalization to other studies trying to identifying genes for hypertension development.

In addition, blood pressure lowering treatment in China is generally low (56.6% in this study), and it would be more likely that treated patients have a more severe hypertension and by that have a stronger link to genetic factors.

Our findings are also supported by the previous study done by Oberkofler and colleagues[6]. They used day time ambulatory blood pressure and defined hypertension as SBP > 140mm Hg or DBP > 90mm Hg or use of antihypertensive medication, and they found a significant association between G482S and hypertension in men[6]. Because the ambulatory blood pressure is lower than occasional clinic blood pressure, their findings were actually in accordance with ours.

Besides, we also found an additive synthetic effect of G482S and +2962A/G genotypes. Although the mechanisms to explain the effect remain unclear, some studies did provide useful evidence. There is substantial evidence for the existence of both monogenic and polygenic forms of essential hypertension, although these are believed to be modulated by both gene-environment and gene-gene interactions[8]. It is well known that both monogenic and polygenic forms of essential hypertension are often early-onset and more severe [3]. As a coactivator of PPAR*γ*, PGC-1*α *regulates PPAR*γ *gene expression which combined with its ligands (thiazolidinediones) to suppress angiotensin II (AII) type 1 receptor (AT1R) gene expression both at the mRNA and protein levels in vascular smooth muscle cells(VSMCs)[4]. Activation of AT1R by AII binding triggers a variety of signal transduction pathways including the mitogen-activated protein (MAP) kinase pathway. AII exerts many biological effects such as cell construction, proliferation, and migration of VSMCs that contributes to the progression of hypertension and atherosclerosis. PPAR*γ *phosphorylation by the MAP kinase pathway may thus attenuate PPAR*γ*-mediated AT1R gene transcription suppression through the inhibition of PPAR*γ *activity[18]. PGC-1*α *is also a coactivator of other nuclear receptors, including thyroid hormone receptor, the mineralocorticoid and estrogen receptors (ER) *α *and *β*, which are also involved in blood pressure control[6-8,19].

Our study also has some limitations. First, there were 32% of none-responders in the study. The main reasons of not attending includes busy at farming work, out to do private small business, not having interest, and not like blood being drawn. The possible bias can not be totally eliminated though no reason was associated with hypertension or biological tests. Second, a significant deviation from the Hardy-Weinberg equilibrium (HW-E) was detected for the +2962A/G polymorphism in our study. This contrasts to the previous study by Oberkofler [6]. The findings that G482S genotypes in the same gene were in accordance with HW-E in both studies may not indicate that the deviation from HW-E was directly due to genotyping error. We retyped these polymorphisms independently using sequencing method in eight participants and ensured no genotyping error occurred. Finally, the possibility of type 1 error due to multiple tests is not eliminated but the systematic difference between regular and severe hypertension for both gene SNPs suggested that is not likely the case.

## Conclusion

In this study, we found that G482S and +2962A/G SNPs of PGC-1*α *gene were only significantly associated with severe hypertension defined by occasional clinic blood pressure measurements. This finding suggested severe hypertension rather than regular hypertension should be used as the outcome in studies on association of genes and blood pressure or hypertension, in order to have a better power.

## Competing interests

The author(s) declare that they have no competing interests.

## Authors' contributions

GX did literature review, performed the statistical analysis and drafted the manuscript. DG and SL organized the field survey for collection of data. YW and YL designed the study, applied for funding and revised the manuscript. All authors read and approved the final manuscript.

## Pre-publication history

The pre-publication history for this paper can be accessed here:


